# Comparative study of stigma and discrimination among vaccinated and non-vaccinated COVID-19 survivors in Bangladesh

**DOI:** 10.1186/s12879-025-10734-8

**Published:** 2025-03-10

**Authors:** Tamanna Rashid, Shamsul Arefin, Mowsume Bhattacharjee, Ashraful Islam

**Affiliations:** 1 Department of Sociology, University of Massachusetts, Amherst, 200 Hicks Way, Thompson Hall, Amherst, MA 01003 USA; 2 Department of Sociology, Gopalganj Science and Technology University, Gopalganj, 8100 Bangladesh; 3 Department of Sociology, Jagannath University, Dhaka, 1100 Bangladesh; 4 Department of Sociology, University of Dhaka, Dhaka, 1000 Bangladesh

**Keywords:** Bangladesh, Comparative case study, COVID-19 survivors, Cultural context, Discrimination, Non-vaccination, Public health measures, Qualitative research, Social stigma, Vaccination

## Abstract

This study investigates the stigmatized experiences of vaccinated and non-vaccinated COVID-19 survivors in Bangladesh, emphasizing the factors contributing to stigma, the lived realities of stigmatization, and strategies for mitigating discrimination. Employing a qualitative comparative case study approach, the research draws on interviews with 22 survivors (13 non-vaccinated and 9 vaccinated) conducted during two critical phases of the pandemic: July–August 2020 and October 2022–April 2023. The findings reveal that stigma and discrimination were pervasive, stemming from deep-seated fears of death and infection, exacerbated by public health measures such as lockdowns, public announcements, and the symbolic marking of houses with red flags. Experiences surrounding COVID-19 testing further intensified stigma, influencing how survivors were perceived and treated by others. Notably, non-vaccinated survivors reported a broader spectrum of stigmatizing experiences compared to their vaccinated counterparts, highlighting the differential impact of vaccination status on social experiences. Despite these challenges, survivors identified key strategies for alleviating stigma, including robust social support, strengthened community connectivity, normalized attitudes toward COVID-19, and proactive media engagement. As a result, this study offers valuable insights for healthcare professionals and policymakers in Bangladesh, informing the development of effective pandemic management strategies. Additionally, it enhances our understanding of the complex interplay between policy, social perception, and individual experiences in shaping pandemic-related stigma and discrimination, highlighting the critical role of community support and care in addressing these challenges.

**Supplementary information:**

The online version contains supplementary material available at 10.1186/s12879-025-10734-8.

## Introduction

The COVID-19 pandemic, characterized by its rapid transmission and complex modes of contagion, has prompted widespread public health concerns worldwide [1, 2]. Early in the pandemic, the global community grappled with the virus’s mutability, its ability to spread rapidly, and the challenges posed by its asymptomatic transmission [3]. However, beyond the biological impact, one of the most significant social consequences has been the stigma and discrimination experienced by those infected with the virus, their families, and related groups, including healthcare workers and immigrants [4]. As the virus spread, individuals were disproportionately targeted based on their perceived association with the disease, leading to verbal and physical abuse, social exclusion, and even eviction [5–7]. This societal reaction was not confined to specific regions but was observed across the globe, exacerbating existing prejudices, discrimination, and xenophobic tendencies [8].

From a sociological perspective, ‘*stigma*’ can be understood as the process through which certain individuals or groups are labeled with negative attributes, leading to social exclusion and discrimination [9, 10]. Stigma, especially in health crises, manifests through processes of labeling, stereotyping, and marginalization based on perceived or actual association with a disease condition, while discrimination is portrayed by the prejudicial treatment of different categories of people as a result of the stigma attached [11, 12]. The Health Stigma and Discrimination Framework identifies three primary forms of stigma: perceived or internalized stigma (how individuals believe they are treated by others), anticipated stigma (the fear or expectation of future discrimination), and enacted stigma (actual experiences of discrimination) [13, 14]. These dimensions of stigma offer a comprehensive understanding of how individuals experience and internalize stigma, particularly in the context of health-related conditions such as COVID-19. Each form can have distinct impacts on individuals’ mental health and social interactions, influencing how they navigate their daily lives and engage with others during and after the pandemic. Those with stigmatized health conditions may face rejection from loved ones, lose employment opportunities, or encounter substandard healthcare services, which can lead to depression, anxiety, or social alienation [15, 16]. Such experiences are not unique to COVID-19 but have been reported in other infectious diseases such as HIV and SARS [14].

Similarly, the COVID-19 pandemic has led to fear, anxiety, and heightened stress levels, contributing to social stigma and discrimination against those affected by COVID19, as well as their family members, close friends, and healthcare providers [17]. In both contexts, stigmatized individuals have concealed their disease status, avoided testing, or refrained from seeking medical care to avoid being stigmatized [18, 19]. These behaviors undermine public health efforts, perpetuate disease transmission, and disrupt effective healthcare policies. During the COVID-19 pandemic, stigma was exacerbated by the rapid spread of fear and misinformation through both social and print media, amplifying societal anxiety and prejudice [4, 5]. These media platforms played a significant role in shaping public perceptions, often highlighting negative narratives about those infected with the virus, which contributed to the social ostracization and marginalization of COVID-19 survivors. As a result, people who tested positive, along with their families and healthcare providers, were often subjected to stereotyping, isolation, and aggressive attitudes [14]. These dynamics reflect broader societal fears, underscoring the need to address stigma not only as an individual issue but as a systemic public health concern. Mitigating stigma is critical for promoting health-seeking behavior, ensuring equitable healthcare access, and effectively managing pandemic outbreaks [20].

The effects of COVID-19-related stigma and discrimination have been most pronounced among healthcare workers, women, slum dwellers, immigrants, individuals of Asian descent, and those diagnosed with the virus. In many Western countries, individuals of East Asian descent were particularly targeted, reflecting pre-existing racial prejudices that were amplified by the pandemic [21]. Healthcare workers, essential to pandemic response efforts, were also stigmatized, not only due to their exposure to the virus but also because they were perceived as vectors for its spread [5]. This stigma significantly hindered public health responses, as those affected by it often avoided seeking care, testing, or disclosing symptoms due to fear of discrimination [22]. In addition to the broader societal stigma faced by COVID-19 survivors, the pandemic also highlighted the differing experiences of stigma based on vaccination status. Vaccination became a critical factor in how individuals were perceived and treated during the pandemic. Those who were vaccinated, particularly those who received full vaccination, were often regarded as less likely to transmit the virus, and thus faced less stigma compared to non-vaccinated individuals. On the other hand, non-vaccinated individuals were often subjected to increased discrimination, as they were perceived as more responsible for the virus’s spread [23, 24]. This division in stigma based on vaccination status emphasizes the complex intersection of public health policies, cultural attitudes toward health behaviors, and societal perceptions of risk. Non-vaccinated individuals, in particular, were more likely to avoid healthcare settings or refrain from seeking testing, further exacerbating transmission [23].

In Bangladesh, as in other parts of the world, the stigma surrounding COVID-19 mirrored these global patterns. The early stages of the pandemic, marked by strict lockdowns and widespread fear, led to significant social exclusion and discrimination against those infected with the virus [25, 26]. In rural areas, individuals who tested positive for COVID-19 often faced abandonment by family members, denial of religious rites, and even refusal of burial from the local people [27]. The gendered nature of stigma was particularly apparent, as women were disproportionately blamed for bringing the virus into their households, exacerbating existing gender inequalities [28]. However, as vaccination campaigns gained momentum, a reduction in fear and stigma was observed, particularly as a large portion of the population received vaccines, leading to a decline in discrimination. The stigma and discrimination faced by COVID-19 survivors and healthcare workers have been widely documented in global studies [15, 16, 22, 29–36] including Bangladesh [37–40]. However, research examining the differential experiences of stigma and discrimination based on vaccination status remains limited, particularly in the context of Bangladesh. Existing studies, including those by Bor et al. [5], Des Jarlais et al. [23], Bardosh et al. [41], Sattler et al. [42], Briciu et al. [43], and Ruiz-Giardin et al. [44], have explored discriminatory attitudes between vaccinated and unvaccinated individuals across various cultural contexts, including the United States, India, Mongolia, Jordan, Romania, and Spain. For example, Bor et al. found that in 19 of 21 Western countries, vaccinated individuals exhibited discriminatory attitudes toward the unvaccinated, with Romania and Hungary being the exceptions. These discriminatory attitudes were more pronounced in cultures with stronger cooperative norms [43]. Similarly, Des Jarlais et al. observed a rise in stigmatization of the unvaccinated in the United States after the introduction of vaccines [23], while Sattler et al. emphasized the need to reconsider vaccination policies in countries like Mongolia, India, and the United States to ensure cultural sensitivity [5]. Despite these valuable contributions, significant gaps remain in understanding how vaccination and non-vaccination status influences lived experiences of stigma and discrimination by the COVID-19 survivors.

While the stigma surrounding COVID-19 has been extensively documented, the differing lived experiences of vaccinated and unvaccinated individuals remain underexplored, particularly in the context of Bangladesh. Globally, some qualitative studies have examined the lived experiences of various groups, including the older adults [37, 45–48]. However, to the best of the authors’ knowledge, no qualitative research has been conducted to investigate how societal norms and structural policies—especially vaccination policies—shape the differential experiences of vaccinated and unvaccinated COVID-19 survivors in various pandemic phases. In Bangladesh, specifically, there is a significant gap in understanding how these factors influence stigma and discrimination faced by survivors based on their vaccination status. This study seeks to address this gap by exploring the role of vaccination status and social and psychological factors in shaping the stigmatization processes experienced by COVID-19 survivors in Bangladesh. Adopting a qualitative comparative approach, this research seeks to provide a comprehensive understanding of how stigma and discrimination function within particular socio-cultural and policy contexts, and how these dynamics evolve over time. By examining different phases of the pandemic and comparing the experiences of vaccinated and non-vaccinated COVID-19 survivors, the study aims to shed light on the shifting nature of stigma in response to both individual and societal factors. The central research question guiding this study is: *How do socio-cultural context and structural policies shape the differing experiences of stigma and discrimination faced by vaccinated and non-vaccinated COVID-19 survivors in Bangladesh*,* and what underlying factors drive these disparities?* Through this lens, the study examines the contributing factors to stigma, the everyday experiences of discrimination, and potential strategies for mitigating stigma.

By addressing these dimensions, the research aims to make a significant contribution to the broader discourse on health-related stigma, particularly in the context of pandemics. It seeks to inform the design of public health policies in Bangladesh that are not only effective but also equitable and culturally sensitive. Recognizing the importance of understanding local socio-cultural dynamics, the study highlights the need for policies that consider the unique experiences of diverse communities. Furthermore, this research aspires to provide actionable insights for mitigating stigma during future public health crises. By emphasizing culturally aware and inclusive strategies, the study advocates for approaches that promote social cohesion and resilience, ultimately fostering more supportive environments for individuals facing health-related discrimination.

### Cultural context, public health measures and approaches during COVID-19 in Bangladesh

In Bangladesh, the social and cultural perception of infectious diseases has historically been shaped by a blend of religious beliefs, cultural norms, and public health policies, which have often led to stigmatization of those affected [49]. Diseases like leprosy and tuberculosis were traditionally seen as divine punishments or moral failings, with sufferers facing social exclusion due to perceived impurity or sin [50]. Religious interpretations often overshadowed medical explanations, as seen during cholera outbreaks, where communities viewed the disease as a punishment from God, leading to reliance on spiritual healing rather than medical intervention [51]. This belief system still permeates attitudes toward diseases like COVID-19, where affected individuals are often blamed for their condition, perceived as negligent or immoral, particularly in rural areas where access to accurate health information is limited. Public health policies, while aiming to control disease spread, have sometimes exacerbated stigma by isolating patients and framing them as threats to public safety, as evidenced during the COVID-19 pandemic when survivors and healthcare workers faced ostracism [25]. The combination of these cultural, religious, and policy-driven factors creates a societal environment where stigma and discrimination thrive, limiting the effectiveness of health interventions and marginalizing vulnerable populations.

The COVID-19 pandemic presented unprecedented challenges to Bangladesh following the first reported case on March 8, 2020 [18]. After that, the then-government, often described by scholars as a “hybrid regime” or “competitive authoritarian,” [52] implemented several public health measures to contain the virus, including a nationwide lockdown from March 16 to May 30, 2020, and the closure of educational institutions from March 16, 2020, to September 11, 2021 [19]. In addition, measures such as shutdowns, strict isolation, suspension of transportation and international flights, mandatory mask-wearing, home isolation, and quarantine protocols were implemented until August 2021 to control the virus’s spread [25, 53]. However, these measures, coupled with restrictions on movement and physical contact as well as inadequate public awareness, created significant societal stress, fueling anxiety and fostering stigma [53]. In certain areas, people protested against these measures due to their negative impact on their financial well-being [54]. Furthermore, the early stages of the pandemic were characterized by widespread misinformation and fear, often exacerbated by sensational media reporting [25, 55]. Various media, particularly social media, initially fueled stigma by spreading misinformation and false narratives, leading to community ostracization and discrimination against COVID-19 patients and their family members [54, 55]. In many places, especially in rural areas of Bangladesh, infected persons and their family members faced eviction, social isolation, and, in extreme cases, denial of burial rights [27].

Government policies, though crucial for pandemic control, unintentionally amplified stigmatization [56]. One such measure involved marking the homes of COVID-19-positive individuals with red flags to enforce isolation. While the intention was to protect public health, this visual indicator became a symbol of fear and alienation in rural villages, fostering societal rejection [37]. The lack of culturally sensitive communication and community involvement in implementing such measures further deepened stigmatization. Concurrently, cultural and religious practices also influenced societal behavior during the pandemic. Reports of communities resisting the burial of COVID-19 victims in local cemeteries or denying last rites demonstrated the intersection of cultural beliefs and public health challenges [38, 39].

**Fig. 1:**
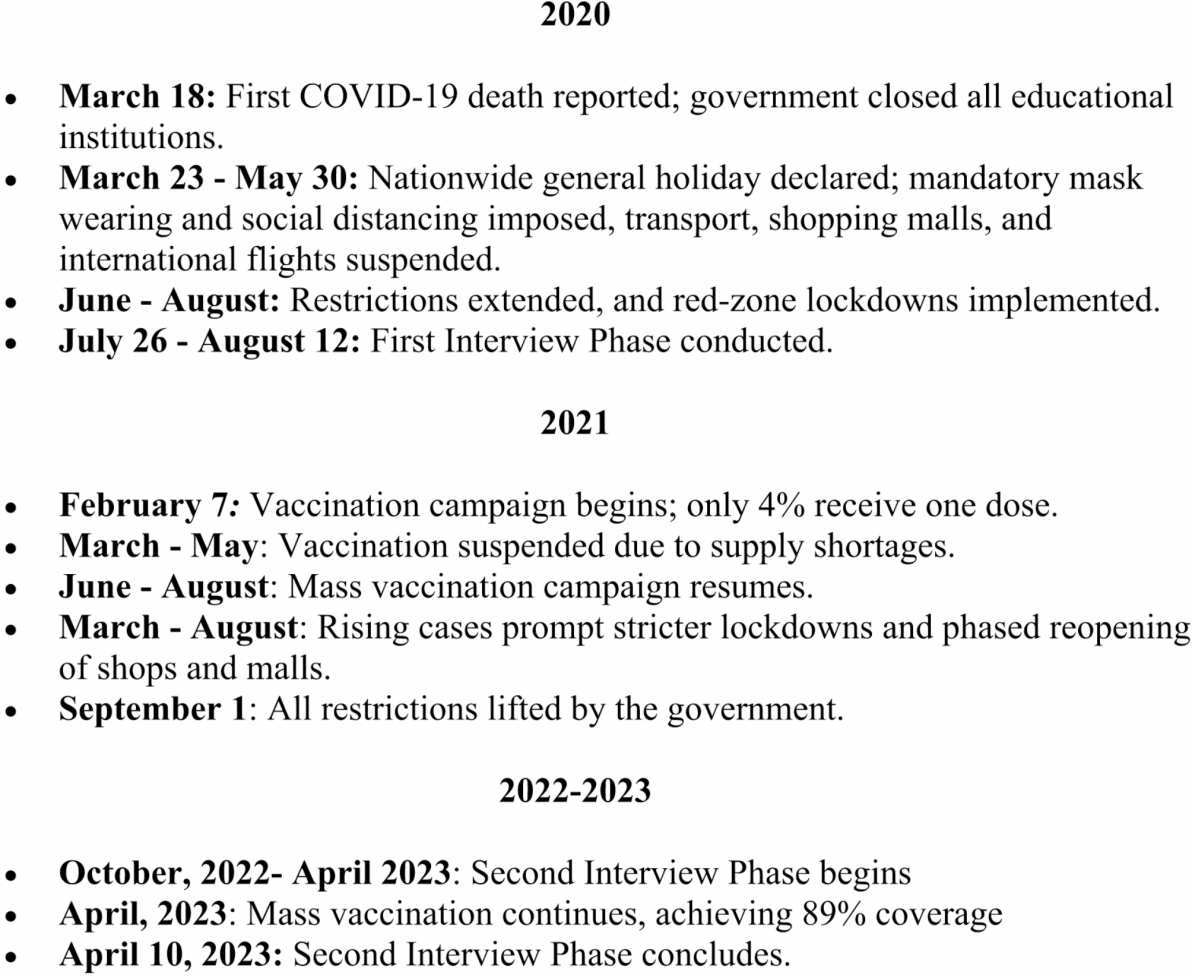
A comprehensive timeline showcasing the implementation of public health measures, the rollout of the mass vaccination program, and the interview phases.

The mass COVID-19 vaccination program in Bangladesh, launched on February 7, 2021, marked a significant milestone in combating the pandemic and reducing associated stigma, despite numerous logistical and political challenges [43, 57, 58]. Initially, the program prioritized frontline workers before expanding to the general population and adolescents. However, its reliance on vaccines like Oxford-AstraZeneca from India faced a major setback when India halted exports during its domestic crisis in 2021. Although vaccine supplies resumed in June 2021, the disruption forced the government to secure alternative sources, including Sinovac, Sinopharm, Pfizer-BioNTech, and Moderna. These delays in procurement and distribution led to public frustration and eroded confidence in the program [59, 60]. Further challenges included vaccine hesitancy, fueled by misinformation and low trust in public institutions [59]. Despite these initial obstacles, the program gained momentum through targeted public awareness campaigns and collaborative efforts. By April 2023, approximately 88.39% of the population had received at least one vaccine dose [58]. The program’s ultimate success significantly reduced infection rates, alleviated public fear, and contributed to a 79% global reduction in COVID-19-related fatalities during the first year of vaccine deployment [24] (see figure-1).

Complementary public health campaigns emphasizing empathy, education, and disease awareness also played a pivotal role in addressing the social impact of stigma. As the pandemic progressed, traditional and social media became instrumental in combating misinformation and fostering solidarity. Campaigns by organizations like BRAC and WHO Bangladesh utilized social media to share recovery stories, counter misinformation, and promote unity. Additionally, health professionals leveraged platforms such as Facebook Live to educate the public and dispel myths. These efforts contributed to a significant decline in stigma-related discrimination and harassment against COVID-19 patients, as reflected in public discourse and media reports [49, 61]. Overall, the vaccination program and associated stigma-reduction strategies demonstrated Bangladesh’s resilience in navigating the complex challenges of the pandemic.

Given the cultural and policy dynamics outlined earlier, it is assumed that individuals who contracted COVID-19 during the early stages of the pandemic faced significantly greater challenges than those infected later, especially as vaccination efforts gained momentum. This study aims to provide a comprehensive understanding of the stigmatization and discrimination processes by comparing the experiences of non-vaccinated and vaccinated COVID-19 survivors at different stages of the pandemic in Bangladesh. In the following sections, we will outline the materials and methods used in this study.

## Materials and methods

### Study design and participants

**Fig. 2:**
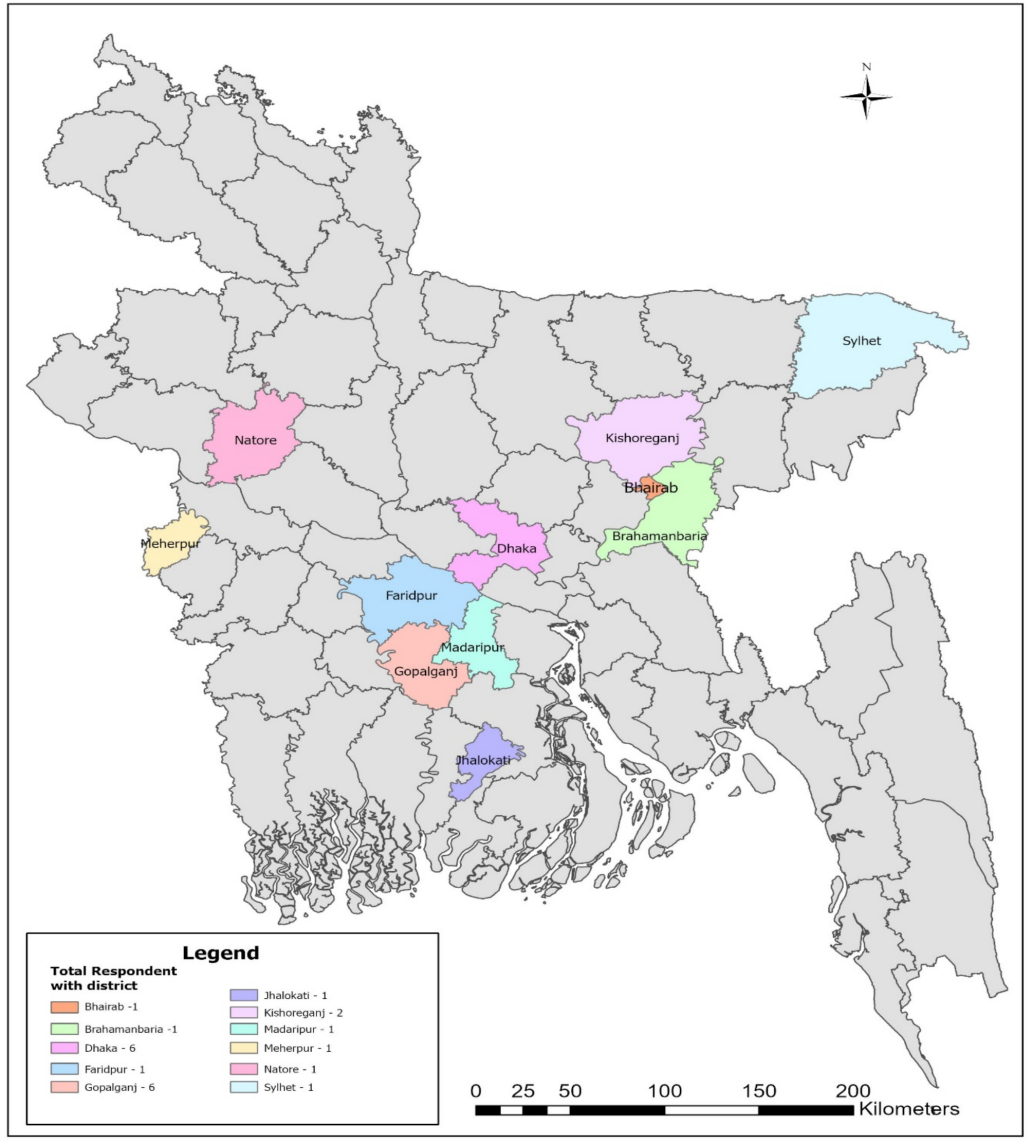
District wise sample distribution for this study (using GIS, this map is developed by safwat sristy)

We conducted a qualitative research using a comparative case study design [62] to investigate the experience of stigma and discrimination faced by non-vaccinated and vaccinated COVID-19 survivors in Bangladesh. Ontologically, the study adopted a constructivist paradigm, recognizing stigma as a socially constructed phenomenon shaped by cultural and contextual factors, while epistemologically, it aligned with interpretivism, emphasizing the understanding of participants’ lived experiences and the meanings they ascribe to them [63]. This study design provides an in-depth understanding of how experiences of COVID-19-related stigma and discrimination have changed over time during the pandemic. We employed a convenient sampling technique to access COVID-19 survivors due to several constraints during the pandemic, such as lockdown restrictions, fear of transmission, and a limited number of available and accessible participants to be included in the study. As a result, the study engaged two distinct participant groups during two separate time periods to examine the evolving experiences of stigma and discrimination among COVID-19 survivors and to assess the role of vaccination in shaping these experiences. To capture a comprehensive understanding of survivors’ lived realities, the study included participants from diverse socioeconomic backgrounds and geographic regions in Bangladesh (figure-2). The sample encompassed individuals from various professional and social categories, including healthcare providers, government officials, business professionals, private sector employees, journalists, students, homemakers, unemployed individuals, and retired senior citizens. This diversity allowed for a nuanced exploration of the intersection between social contexts and the experiences of stigma and discrimination over time.

The inclusion criteria for both non-vaccinated and vaccinated participants were: (1) individuals who tested positive for COVID-19 through clinical testing, and (2) individuals who were quarantined for a minimum of two weeks, either at home or in a hospital setting. The only distinction was that vaccinated participants had received at least two doses of the COVID-19 vaccine before contracting the virus. All participants voluntarily consented to participate in the study. A total of 22 respondents were interviewed, comprising 13 non-vaccinated and 9 vaccinated survivors (see Table-1). The sample size was determined based on data saturation, when no new themes emerged during the interviews.

**Table 1:** Characteristics of the respondents (*N* = 22)

Code	Gender	Age	Occupation	Residence	Isolation status	Vaccination
R1	F	22	Student	Rural	16	No
R2	M	22	Student	Urban	14	No
R3	M	25	Private job	Urban	25	No
R4	M	23	Student	Rural	12	No
R5	M	29	Police	Urban	36	No
R6	F	32	Magistrate	Urban	14	No
R7	M	35	Businessman	Rural	14	No
R8	M	30	Banker	Urban	14	No
R9	M	37	Businessman	Rural	28	No
R10	F	30	Banker	Rural	14	No
R11	F	28	Housewife	Urban	20	No
R12	M	76	Retired politician	Rural	18	No
R13	M	31	Physician	Urban	18	No
R14	M	33	Private job	Urban	16	Yes
R15	M	32	Journalist	Urban	14	Yes
R16	F	21	Student	Rural	14	Yes
R17	F	21	Student	Rural	16	Yes
R18	M	32	Physician	Urban	14	Yes
R19	M	36	Private job	Rural	18	Yes
R20	F	30	Faculty	Urban	14	Yes
R21	F	27	Journalist	Urban	14	Yes
R22	M	27	Police	Rural	14	Yes

### Interview outline

We aim to understand how COVID-19 survivors perceive their personal experience of contracting COVID-19 disease in the context of stigmatization and discrimination. Hence, we employed an in-depth interview approach. This method allows participants to narrate and describe their experiences with greater detail as they have lived through COVID-19 disease-related experiences and vulnerabilities. An interview checklist (attached as supplementary file) was prepared based on the published literature and reviewed through several meetings with the research team (TA, SA, MW, MAI). The interview guideline includes the following broad sections with key points to understand the experiences of COVID-19-related stigma and discriminations: (1) Participants’ perspective and understanding of COVID-19. 2) Participants’ experiences of earlier symptoms, testing procedures, and vaccination. (3) Law enforcement agency’s initiatives (lockdown, putting red flags, etc.) and social vulnerabilities. 4) Experience of quarantine and isolation. 5) Experiences of stigma and discrimination: social, economic, and mental. 6) Respondents’ feedback on the support they needed and suggestions to minimize stigma and discrimination.

### Data collection

We adopted a qualitative approach using in-depth interviewing to elicit relevant information from the respondents. The data collection happened in two different phases of the pandemic with two different group of participants. In both cases, we employed the same interview guidelines mentioned above for two distinctive groups. In the first phase, we interviewed 13 non-vaccinated survivors between July 26 and August 12, 2020, during the first wave of the pandemic in Bangladesh. In the second phase, we interviewed 9 vaccinated participants between October 10, 2022, and April 10, 2023 (see figure-1). The reason we collected data in two time periods was to compare the differential experiences of stigma and discriminations faced by both survivors.

In both phases, we identified the respondents through several means, such as a social media campaign and using personal networks. The interviews were conducted via telephone and online platforms, including Zoom and WhatsApp, in accordance with ethical guidelines to prevent contributing to the spread of the virus. Initially, we scheduled 15 non-vaccinated respondents but stopped after 13 interviews since data saturation was achieved thereafter, making further interviews unnecessary. During the second phase, we encountered challenges in recruiting vaccinated participants as many people were less interested in taking part in COVID-19-related research. This may be attributed to decreased concerns about the virus and the return to normal life following an extended period of lockdown. After running several social media campaigns and leveraging personal networks, we successfully interviewed 9 vaccinated respondents who willingly took part in the study.

The researchers provided the participants with an overview of the study’s context and purpose and obtained their verbal consent to take part. Additionally, most participants were emailed a consent form and interview guide. Upon agreeing to be interviewed, participants had the option to schedule telephone or Zoom interviews at their convenience. The interviews, conducted in Bangla by four contributing authors (TR, SA, MB, MAI), ranged from 20 to 65 min in duration. All interviews were audio recorded with the participants’ informed consent using an electronic device.

### Data analysis

We employed thematic analysis for its systematic yet flexible approach in identifying patterns within qualitative data, making it ideal for exploring the stigma experiences of vaccinated and non-vaccinated COVID-19 survivors in Bangladesh [64]. This method allowed us to compare shared and divergent stigma experiences between the two groups, which would have been more challenging with methods like narrative analysis, grounded theory or phenomenology [62]. The flexibility of thematic analysis also enabled us to incorporate verbatim excerpts, ensuring authentic representation of participants’ voices while providing rich contextual insights. We maintained credibility by resolving coding discrepancies through consensus and validating findings in follow-up discussions with participants. Additionally, thematic analysis helped uncover underlying social processes such as discrimination, which made it particularly effective in capturing the complexity of stigma within its socio-cultural context [65].

Initially, we transcribed the audio recordings in Bangla to fully capture participants’ expressions before translating them into English. Each translated transcript was cross-verified by another team member who compared the English version with the original Bangla to identify any inconsistencies, unclear phrases, or potential loss of meaning. A significant challenge was balancing literal translation with cultural fidelity, especially for phrases without direct English equivalents, requiring interpretive judgment and consensus among team members. To ensure rigor in both translation and analysis, we consulted a qualitative research expert. These steps align with established practices in multilingual qualitative research, emphasizing meaning over literal translation and collaborative cross-verification to enhance reliability [66].

Each team member reviewed the transcripts multiple times to familiarize themselves with the content and identify recurring themes. We then developed a preliminary coding framework using Microsoft Excel 365, refining it through several team discussions. The themes were generated by focusing on patterns, similarities, and differences in stigma experiences between vaccinated and non-vaccinated individuals. To ensure consistency, each transcript was coded by at least two team members, and any discrepancies were resolved through consensus.

While our thematic analysis followed an inductive approach, treating the data holistically to allow themes to emerge organically, distinct patterns linked to vaccination status became evident during the interpretation phase. For instance, non-vaccinated participants often described stigma stemming from public health pressures and social expectations, whereas vaccinated participants reported experiences tied to skepticism or the need to justify their decisions. These distinctions were revisited and refined during analysis to ensure that findings were rooted in the data, aligning with best practices in qualitative research [64, 65]. This iterative process balanced emergent themes with a targeted focus on vaccination status, ensuring transparency and a nuanced understanding of stigma within its socio-cultural context. The findings were organized into three major themes and twelve sub-themes (see table-2) supported by verbatim excerpts to ensure participants’ perspectives were accurately represented. Where ambiguities arose, we held follow-up meetings with participants to clarify their intended meanings.

**Table 2:** Themes and Sub-themes

Themes	Sub-Themes
Contributing factors to stigmatization and discrimination	*Fear of death*
	*Fear of infection*
	*Public health measures: lockdown*,* public announcements*,* and putting red flags*
	*COVID-19 testing exacerbates the process of stigma*
Experiences of stigma and discrimination	*Labeling and blaming*
	*Social disconnectedness and ostracization*
	*Non-cooperation from colleagues and close ones*
	*Internalized stigma*
Strategies to combat stigma and discrimination	*Social support and connectivity*
	*Corona as a treatable disease*
	*Access to testing and mass vaccination*
	*Monitoring news media*

## Results

### Characteristics of the participants

The study included participants who had tested positive for COVID-19 through clinical testing. Of the participants, 64% (14) were male and 36% (8) were female, with an average age of 30 years. The objective of this study is to examine the stigma and discrimination faced by COVID-19 survivors in Bangladesh. To achieve this, respondents from diverse socio-economic backgrounds were included, rather than focusing on any specific social group. In order to enhance the overall inclusivity of the research findings, deliberate efforts were made to ensure representation from both rural (54.54%) and urban (45.46%) areas. Approximately 46% of the respondents were frontline workers, such as physicians, police officers, magistrates, bankers, and journalists. This group faced greater susceptibility to stigma and discrimination due to their professional roles during the pandemic. Additionally, 27% of the respondents were students who faced challenges at their educational institutions and social life during COVID-19. All respondents underwent quarantine and follow isolation for a period of 12–36 days. Furthermore, 80% of the participants practiced home quarantine, while the remaining individuals were admitted to specialized hospitals dedicated to COVID-19 treatment.

### Theme I: contributing factors to stigmatization and discrimination

During the COVID-19 pandemic in Bangladesh, the stigmatization and discrimination of individuals, both non-vaccinated and vaccinated, were significantly shaped by widespread fear of infection and death. This fear was amplified by intense media coverage and strict government measures, including lockdowns, public announcements through miking, and marking infected households with red flags. Additionally, the social stigma surrounding COVID-19 testing and disclosing a positive status further heightened social vulnerabilities. These dynamics were observed through interviews conducted during two distinct periods: July–August 2020 and October 2022–April 2023. Now, important findings are discussed below under the following sub-themes:

#### Fear of death

In this study, the majority of non-vaccinated respondents stated that they became afraid after being identified as COVID-19 positive. They started to believe that their life is in danger due to this disease. According to the respondents, the repeated and widespread news about COVID-19 infections and the severity of the disease, both globally and in Bangladesh, frequently broadcast on television and shared on social media, intensified the fear of death among people, including those infected with the virus. Consequently, some participants mentioned that they avoided using social media during their quarantine period to manage stress and maintain calmness. Moreover, individuals infected with COVID-19, those showing symptoms, and their family members experienced avoidance and neglect due to the widespread fear of death among the general population. During an interview with a non-vaccinated respondent who was a police officer, he vividly described the intense fear of death that gripped people during the early stages of the pandemic in Bangladesh. According to him:

“When I heard that I was diagnosed as COVID-19 positive, I felt like I would die very soon. I was completely broken inside and felt depressed. This was during the very early stages of the pandemic in Bangladesh, when we were first hit. We had access to all the information, data, and reports about the severity of COVID-19 in developed countries. We also learned that even those countries, despite having advanced medical technology, couldn’t manage the situation properly. On top of that, I was receiving daily updates from television and Facebook about the potential consequences in Bangladesh. All this information felt very threatening, and the lack of logistical support to fight the disease made it even worse for me.” *(R5*,* Police*,* Non-vaccinated)*.

In contrast, a small number of vaccinated individuals reported experiencing a fear of death when they contracted the virus during the second phase of pandemic. The majority of them remained positive about their recovery and were not much worried about disease. They expressed confidence in their ability to recover due to their immunization. In addition, print and social media telecast less news on COVID-19 deaths and the death rates were much lower than the earlier stages. A few respondents mentioned being motivated by survivors’ stories shared through Facebook posts during the later phases of the pandemic, which they believed helped reduce the public’s fear of death. During the interviews, we spoke with several vaccinated individuals who had contracted COVID-19 two or more times. These individuals were able to clearly distinguish the differences in experiences during the later phases of the pandemic. As one respondent explained:

“When I first got infected in August 2020, I was really scared. But this time, being infected for the second time, I wasn’t as fearful because I was vaccinated. It did surprise me, but I had already learned from social media that vaccinated people could still get infected. Another thing is that the coronavirus isn’t as dangerous as we initially thought. With the government’s mass vaccination program, almost everyone around me was vaccinated, so I had less fear of death this time.” *(R19*,* Businessman*,* Vaccinated)*.

#### Fear of infection

Both non-vaccinated and vaccinated participants in the study expressed anxiety and concern about potentially spreading the virus to their loved ones, despite their own confidence in their ability to recover. Many also reported feeling guilty about the possibility of infecting their family and friends. To minimize the risk of transmission, the majority of participants chose to maintain strict isolation at home from their family members and close ones, even though this impacted their mental well-being. The fear of spreading the virus caused feelings of loneliness, alienation and isolation among both vaccinated and non-vaccinated individuals. Respondents shared that the fear of transmitting the virus through social interaction caused panic among their family members, often resulting in the infected person being isolated and feeling alienated. One of the non-vaccinated frontline workers, a banker, shared his feelings during the interview:

“I wasn’t afraid, but I was a little tense because my wife could get infected through me. That’s why I isolated myself. I didn’t have many worries about myself.” *(R8*,* Banker*,* Non-vaccinated)*.

Although the fear of infection was comparatively lower among vaccinated participants, our findings show that it still remained significant during the second phases of the pandemic. In most cases, participants were particularly concerned about their aged parents, as they believed they were most vulnerable to severe effects of COVID-19. As a result, they took extra precautions to ensure their parents’ safety and protection. One vaccinated student shared:

“When the doctor instructed me to undergo a COVID-19 test, a feeling of guilt and fear arose in me. I worried about what would happen if it was actually COVID-19 and someone got infected because of me. My biggest concern was whether my parents might fall sick—my mother has respiratory problems, and my father has undergone open-heart surgery and had a previous heart attack. For me, going home felt like a risky decision because they might somehow get infected through me. It was a mentally overwhelming situation that kept me in a constant state of distress. The mental tension of potentially being responsible for infecting others overshadowed any concerns about my own physical illness.” *(R16*,* student*,* vaccinated)*.

Another vaccinated respondent shared how she faced stigma from her own illiterate fathers due to his fear of infection, which significantly affected her mental health. Despite the emotional toll, she chose to accept the situation and focused on finding ways to cope with it. Reflecting on her experience, she stated:

“Ever since I contracted the Corona infection, my father has been distancing himself from me. While my mother always made sure I had everything I needed and was constantly there for me emotionally, my father kept physical distance. It was painful for me, but I made an effort to accept it casually.*” (R17*,* Student*,* Vaccinated).*

#### Public health measures: lockdown, announcements (miking), and putting red flags

In the early stages of the pandemic, the stigmatization experienced by infected individuals and their families worsened when they publicly identified as coronavirus patients. A large number of non-vaccinated COVID-19 survivors expressed that revealing their infection status made them feel vulnerable, leading to significant stigma from neighbors, relatives, and, in some cases, even their own family members. Additionally, they mentioned that the government’s public health measures including lockdown policies, public announcements (*mikings*), red flags placed in front of infected individuals’ houses, and other measures, heightened the fear and stigma, causing widespread panic. As a result, individuals showing COVID-19 symptoms were hesitant to seek clinical testing and disclose their test results with others. During an interview, one respondent who was a private employee shared how he isolated himself completely outside his home due to the fear of stigma triggered by the government’s initial lockdown policies. According to him:

“I panicked as I provided the police all of my information. If police officers came to my neighborhood and placed a lockdown on my home, everyone would recognize me as the first Corona patient in my neighborhood and be afraid of me. This was quite distressing for me. " *(R3*,* Private job*,* Non-vaccinated).*

Another non-vaccinated high-ranking official of the Bangladesh government shared similar experiences of stigmatization due to the lockdown initiatives. However, she also mentioned that due to her social status and class position, she faced less social stigma than others. In her words:

“The building where I live was completely locked down, and I faced some social stigma. After I got sick, I heard that many officers who worked with me were very scared. Many of my colleagues wanted to distance themselves. However, I believe that due to my social status, I faced fewer social vulnerabilities compared to other COVID-19 sufferers.” *(R6*,* Executive Magistrate*,* non-vaccinated)*.

Moreover, several non-vaccinated participants shared that they felt intimidated, heard negative remarks, and were socially confined to due to public announcements made by security agencies. As a result, despite experiencing coronavirus symptoms, some non-vaccinated individuals chose to conceal their symptoms and avoid getting tested. One of the respondents mention us:

“Hearing the voice on the microphone at midnight, the neighbors in our locality woke up. They became curious about what had happened with us. I also became nervous after hearing the repeated announcements. Additionally, I heard some negative comments from our relatives, which hurt us and made us feel psychologically vulnerable.” *(R4*,* student*,* non-vaccinated)*.

Vaccinated respondents, on the other hand, did not experience measures such as lockdown, miking, and putting red flags on patients’ houses. Hence, they did not experience a similar social process of stigmas to non-vaccinated participants. Moreover, most of them did not experience any difficulties in exposing corona-positive status to others, demonstrating a cooperative and accepted social circumstance for corona-infected patients. According to one vaccinated physician:

“When corona arrived, I was treating patients at a COVID-19 dedicated hospital in *Kurmitola*, Dhaka. It was a difficult time then. But now, everyone knows that corona is treatable. The government’s vaccination program and other measures have reduced fear. So, when I got infected, I informed my colleagues and relatives for their safety. After they learned about my illness, a few of my neighbors even provided food for me.” *(R18*,* Physician*,* Vaccinated)*.

#### COVID-19 testing exacerbates the process of stigma

Almost all the vaccinated and non-vaccinated respondents acknowledged that clinical testing for COVID-19 has resulted in an increase in stigmatization and discrimination. Specially, non-vaccinated participants faced stigmatization and exclusion from their colleagues and acquaintances when they identified COVID-19 after the clinical test. This led them to recognize that coronavirus testing may have exposed them to social vulnerability. During the interview, one of the non-vaccinated respondents, who had previously helped his villagers by spreading knowledge about the coronavirus and was later identified as a COVID-19 patient, couldn’t hide his frustration. He expressed:

“Then it seemed to me that it would be better if I avoided getting tested and remained ‘negative.’ I told myself, ‘I’ve stayed in the house for 3 or 4 months, so I should be able to stay for 14 more days without a test. I should live a normal life like everyone else” *(R4*,* Student*,* Non-Vaccinated).*

Another non-vaccinated respondent, who lived in an apartment in the capital city of Bangladesh, Dhaka, shared how he avoided COVID-19 testing after realizing that it would stigmatize him and possibly result in losing his accommodation. He remarked:

“I was not interested in getting tested at any private hospital or having a sample collected privately because if they came here to collect the sample, the building members would kick me out. And if they found out that I was positive, they would also get me out of here.” *(R3*,* Private job*,* Non-vaccinated).*

On the other hand, most of the vaccinated respondents we interviewed expressed their willingness to undergo COVID-19 testing to help combat the virus’s transmission. They also did not experience much stigma due to their testing. One respondent, a faculty member, shared that although he did not exhibit any physical symptoms of COVID-19, he got tested to ensure the safety of his family members. According to him:

“I felt completely fine from the start and had no physical symptoms of COVID-19. However, I decided to take the test to ensure I was protecting my older parents.” *(R21*,* Faculty*,* Vaccinated)*.

We also found that during the second and later phases of the pandemic, when vaccination was available to almost everyone and the infection rate was higher, many people became reluctant to get tested, as most of them were showing visible symptoms of COVID-19. Consequently, they faced less stigma from others. In response to the question why people avoided testing, one of the students said to us:

“The second time, everyone was infected with corona. There was reluctance to get tested among the patients, so they didn’t realize they were infected. This was one of the reasons they felt less stigma.” *(R17*,* student*,* vaccinated)*.

### Theme II: experiences of stigma and discrimination

Both non-vaccinated and vaccinated COVID-19 survivors experienced significant stigma and discrimination from their relatives, neighbors, colleagues, and close ones when they were isolated for an extended period of time. However, it was found that non-vaccinated survivors reported more cases of stigma and discrimination compared to vaccinated during their isolation. During this period, they experienced various forms of stigma and discrimination, including labeling and blaming, social ostracization and disconnectedness, lack of cooperation from colleagues, and internalized stigma. Such experiences are elaborated in the following sub-themes:

#### Labeling and blaming

During the early stages of the pandemic, non-vaccinated COVID-19 survivors and their families experienced a range of negative stereotypes, including labeling, blame, social stigma, and neglect from neighbors and employers. These attitudes significantly reduced social support and cooperation during their quarantine, ultimately impacting their mental health and physical well-being. Even after recovering and returning to regular activities following quarantine, many participants experienced frequent negative comments from neighbors, house owners, and employers. One individual reported that he and his family faced severe mistreatment from local villagers, including the placement of thorns in front of their door and surrounding their homes with barbed wire to prevent them from leaving. Furthermore, many respondents mentioned that their neighbors and employers lacked empathy and rarely showed any concern for their wellbeing. Instead, many of them exhibited a distressing attitude towards those infected with COVID-19. As one respondent described:

“When my brother and I tested positive for the virus, the district health officer called us. He was shouting and blaming my brother for the entire incident. He kept saying to my brother, “You have brought this disease to this area.” His words greatly upset my brother. The unexpected phone call also surprised me. He behaved in a very insensitive manner. In such a situation, he should have shown sympathy towards my brother’s family instead of blaming him for everything.“*(R3*,* student*,* non-vaccinated).*

Another non-vaccinated respondent, a businessman residing in a village during his infection, reported experiencing similar stigmatizing treatment from his neighbors. Additionally, he faced rejection from his own family members, which had a profound impact on his mental health. The denial by his own family highlights the complex interplay between personal relationships and social stigma, revealing how fear and lack of awareness can erode familial support systems. According to him:

“After my Corona infection, relatives and neighbors started to treat us like enemies. Even my sister, who came and kept food in front of my house and then left, did not visit us. I always had good relations with my neighbors, but during my hard times, they treated us horribly. Truly, we experienced the fear of death on earth before death.” *(R7*,* Businessman*,* Non-Vaccinated)*.

Even in the early stages, some villagers, hospital staff, and neighbors treated COVID-19 survivors as if they were criminals or sinners due to their infection. Some survivors developed the belief that their illness was a punishment from God for their sins. They faced harsh reactions from their hospital staff and neighbors, who blamed the patients for spreading the disease in their community and endangering everyone’s lives. One of the non-vaccinated participants, a police officer who received treatment at a COVID-19 dedicated hospital in Dhaka described his experience as follows:

“When I was kept in isolation at the hospital, nobody came to visit me. One of the nurses shouted at me and said, ‘You brought a very bad disease to our hospital. How can you expect good care?” *(R5*,* Police*,* Non-vaccinated)*.

In contrast, vaccinated COVID-19 survivors in the later stages of the pandemic experienced less stigma, such as blame and labeling. Instead, many reported receiving support and positive responses from their neighbors and loved ones. For example, a vaccinated police officer shared how his neighbors cared for him, even providing food during his recovery. Similarly, a journalist described how his neighbors’ treatment of him changed upon his release from the hospital, attributing this support to his profession. He explained:

“When I returned home from the hospital, the house owner and neighbors didn’t even ask about my illness. It was as if they knew nothing about me. I think perhaps this was because of my occupation.*” (R21*,* Journalist*,* Vaccinated)*.

A vaccinated faculty member living in Dhaka, in an apartment with limited neighbor interaction, reported experiencing less stigma and labeling. Living in an urban setting, where social interactions are less frequent, shielded her from the stigmatization commonly faced by those in closer-knit communities. This contrasts with rural or densely populated areas in Bangladesh, where frequent interactions often lead to heightened stigma. Her experience underscores how the social dynamics of urban living can reduce the stigma associated with COVID-19. According to her:

“I faced less stigma because I was in Dhaka in a flat and we have less interaction within the apartment. That is one of the reason I faced less stigmatized attitude from my neighbors” *(R20*,* Faculty*,* vaccinated).*

Most of the vaccinated participants did not face such negative stereotypical attitudes. However, one student living in a rural village wanted to disclose her illness to the neighbors, but her mother was reluctant to reveal it due to the fear of being stigmatized and labeled by the neighbors. According to her:

“I wanted to disclose my illness to my neighbors and relatives who were coming to visit me, letting them know that I was sick. However, my mother didn’t want others to know about my illness. The reason was that a few months ago, one of our neighbors got COVID-19, and because of that, he was isolated by the neighbors.” *(R17*,* Student*,* vaccinated)*.

This suggests that, although vaccinated participants generally experienced less stigmatization from neighbors and others, in rural areas, a few still recognized the consequences of such negative labeling. As a result, their parents chose to hide their illnesses from the neighbors.

#### Social disconnectedness and ostracization

During the initial phase, non-vaccinated COVID-19 survivors and their family members experienced social disconnection and ostracism from their close relatives and neighbors. The local administration’s implementation of various lockdown measures, including marking the homes of affected individuals with a red flag, inadvertently made them more susceptible. For example, one individual shared that after testing positive for COVID-19, his neighbors and even his sister cut off all contact with him. The fear of infection and stigma was so strong that people avoided visiting the pond at the home of an infected individual, where they had been bathing for years. Furthermore, family members of infected individuals were restricted to attending mosque prayers, and some faced resistance from relatives and neighbors when organizing funerals for the deceased. One respondent from the rural village, who lost his father to a COVID-19 infection, described the challenges they faced due to the circumstances surrounding the death. According to him:

“When my father passed away, none of our neighbors visited our home. We couldn’t find anyone to help carry the casket. Only a few relatives stepped forward to assist us, and we completed the funeral quickly.*“(R7*,* Businessman*,* Non-vaccinated).*

Another non-vaccinated respondent revealed the indirect impact of her COVID-19 infection on her family, as her elder sister’s son was excluded from playing with other children. This exclusion reflects the widespread fear and stigma surrounding COVID-19, where even close family members face social isolation due to perceived contagion risks. It highlights how the social consequences of the virus extend beyond the infected individual, affecting the social lives of their loved ones and reinforcing stigma within the community. She described it as follows:

“One day, I saw my niece crying. I asked her why she was crying, and she replied that she had been denied the chance to play in the field because of my illness.” *(R1*,* Student*,* non-vaccinated)*.

In addition, one of the physicians mentioned how he was socially ostracized by his neighbors because of his profession and was later completely avoided after contracting the infection. According to him:

“As a front-line worker, we had to provide direct service to COVID-19 patients. When I returned home from the hospital, I could sense that they feared me. But when I and four other members of my family got infected with the COVID-19 virus, they completely avoided us. We didn’t receive any support from them during those days.” *(R13*,* Physician*,* Non-vaccinated)*.

Vaccinated patients, in contrast to non-vaccinated individuals, reported fewer instances of stigma and social isolation. Many respondents noted that the confidence gained from vaccination led them to believe that COVID-19 would not have severe consequences for them. This belief often resulted in a more relaxed approach to quarantine, with some vaccinated individuals not strictly isolating themselves. As a result, they maintained closer interactions with family members and close friends throughout their illness, receiving emotional and practical support. According to one of them:

“During the initial stage, almost everyone was terrified of COVID-19. Anyone infected with COVID-19 was considered a criminal. Society looked down on them in a way that could worsen their sickness. However, the vaccination program, government support, and the influence of social media brought a significant change in society. People infected with COVID-19 in 2022 were treated differently than those in 2021; by then, everyone was extremely sincere.” *(R16*,* Student*,* vaccinated)*.

#### Non-cooperation from colleagues

Among the respondents, professionals experienced a mix of cooperation and non-cooperation from their colleagues and employee organizations during their quarantine periods. Specifically, many non-vaccinated professional patients reported experiencing unfriendly behavior and a lack of support from their colleagues, especially during the early stages of the pandemic. Additionally, some colleagues attempted to avoid them in professional settings, further contributing to feelings of isolation and stigma. This lack of cooperation highlighted the challenges that non-vaccinated professionals faced in maintaining their professional relationships and social connections during their illness. One non-vaccinated participant, a police officer who was admitted to a hospital after contracting COVID-19, shared how he and others were treated in the isolation unit by healthcare providers and how this affected their mental health. According to him:

“During our 22 days of hospitalization, no doctors, nurses, or other medical staff ever came to visit us physically, except in some critical circumstances. Instead, they communicated with us over the phone and provided only virtual advice. This seemed to be a very unfriendly approach from the hospital authorities. It frustrated and hurt me a lot, thinking that this was happening to us simply because we were COVID-19 patients.” *(R5*,* Police*,* non-vaccinated).*

On the other hand, most of the vaccinated professionals informed us that they receive good care and cooperation from their colleagues and employee organization. Several professionals shared us that their organization provided them necessary leave, and social support during their isolation. Only one vaccinated professional respondent, who contracted COVID-19 multiple times, reported experiencing non-cooperation and unfriendly treatment from his colleagues and employer organization. As a result, he was compelled to leave his job and start a business, which has led to a substantial financial burden due to the impact of COVID-19. According to him:

“I had to leave my job after my third COVID-19 infection because of the stress from my office. It was my first job, and I continued working there until the third infection, but I left after that. After several bouts with the virus, my colleagues became unfriendly with me. They no longer interacted with me in a friendly manner and maintained distance. When I realized this, I left my job willingly**”***(R19*,* Businessman*,* Vaccinated).*

#### Internalized stigma

Both vaccinated and non-vaccinated COVID-19 survivors experienced a sense of internalized stigma during their recovery and post-recovery phases. At the onset of the pandemic, some respondents noted that the fear of infection contributed to their feelings of internalized stigma. Additionally, several non-vaccinated respondents shared that, during the post-recovery period, they felt unwelcome in social spaces, leading them to avoid public areas. One female respondent specifically mentioned that she refrained from taking the COVID-19 test because she internalized her gender identity and social position within rural society. According to her:

“I avoided the test for two reasons. First, testing is limited in our area, with only two people allowed to test each day, which discourages others from doing it. Second, for women, taking the test is stigmatized in our community.” *(R1*,* student*,* non-vaccinated)*.

This account illustrates how deeply ingrained gender-based stigma and internalized societal norms influence women’s decisions, particularly in rural Bangladesh. The respondent’s hesitation to seek testing highlights the compounded barriers women face—both the logistical limitations of limited testing access and the societal pressure that stigmatizes women who seek medical attention. In this context, women are more likely to internalize these negative perceptions, leading to self-stigmatization and avoidance of necessary health interventions. Such gendered stigma not only exacerbates women’s social isolation and mental health challenges but also perpetuates existing gender inequalities, ultimately hindering public health efforts.

In contrast, some vaccinated participants also reported feeling “*othered*” during post-recovery. One faculty member informed us that she felt uneasy and hesitant during her post-recovery and therefore avoided social gatherings despite her colleagues being supportive. This reflects the lingering psychological effects of stigma, where survivors, despite social acceptance, may struggle with internalized feelings of alienation and anxiety, highlighting the need for ongoing emotional support during reintegration. According to her: “I felt uncomfortable despite being fully recovered, although people were welcoming and at ease with me.” *(R20*,* Faculty*,* Vaccinated)*.

### Theme III: strategies to combat stigma and discrimination

During the pandemic, both vaccinated and non-vaccinated participants faced various forms of stigma and discrimination resulting from COVID-19 infection. These experiences significantly affected their physical and mental well-being, while also exacerbating economic and social vulnerabilities. However, participants also demonstrated resilience by developing various strategies and mechanisms to combat stigma and discrimination during their recovery and post-recovery periods. This section presents effective strategies derived from participants’ feedback, organized under the following sub-themes.

#### Social support and connectivity

Both vaccinated and non-vaccinated individuals shared that they received strong social support from family, relatives, colleagues, and friends, which helped them to recover. Non-vaccinated COVID-19 survivors mentioned the care and encouragement they received from their loved ones during isolation, including mental support, meal preparations, getting essential items, and staying in touch. Some participants also mentioned that their parents didn’t strictly follow isolation rules to make them less alone. This support was crucial during the difficult time. Additionally, married individuals emphasized the unwavering support from their spouses, which kept them mentally strong. An older adult and retired politician, 73, from a rural village shared how his wife constantly cared for him during his isolation, helping him get through the challenge. According to him:

“My wife took care of everything for me, from cooking to providing whatever I needed.*” (R13*,* retired politician*,* Non-vaccinated)*.

Most non-vaccinated survivors who were professionals received significant support from their colleagues, employee organizations, and friends, which greatly aided their recovery. One survivor, who was hospitalized in isolation, shared that the psychological support from colleagues played a key role in his recovery. A government official said:

“We were four colleagues staying the same unit in the hospital, which was a great support for all of us. Whenever we felt down, we would talk, joke, and share our struggles while maintaining social distance. This really helped us recover from the psychological challenges and deal with our situation more effectively. It had an incredible impact that wouldn’t have been possible if I had been alone in a room.*” (R5*,* Police*,* Non-vaccinated)*.

On the other hand, vaccinated COVID-19 survivors in this study highlighted that social support and consistent communication were essential for their mental well-being. Even during quarantine, they remained connected with family, friends, colleagues, and acquaintances. This network and communication played a key role in reducing stigma and discriminatory attitudes toward COVID-19 patients. During a pandemic, when physical contact is limited, emotional support through communication becomes invaluable. A medical professional said:

“At that time, the most important thing was that we talked a lot. I belonged to a large and well-connected family. Despite being in isolation, we stayed connected with each other. My relatives and friends constantly cared for us and asked if we needed anything. They were also great sources of support and courage.*“(R18*,* Physician*,* Vaccinated).*

#### Corona as a treatable disease

One significant distinction between vaccinated and non-vaccinated participants, which also influenced their recovery, was their perception of COVID-19. During the early phase of the pandemic, COVID-19 was widely seen as a deadly disease. Government measures such as closing educational institutions, enforcing strict lockdowns, public announcements, marking infected houses with red flags, limited testing kits, lack of vaccines, stereotyped media coverage, and a general lack of public understanding contributed to this perception. As a result, almost all non-vaccinated survivors reported feeling panicked when they were infected, fearing death and the possibility of spreading the virus to others. While most initially believed COVID-19 was untreatable, a few participants—primarily students—viewed it as a treatable disease. A 21-year-old female student, who was non-vaccinated and actively worked to raise awareness in her village about COVID-19, shared her experience of being infected:

“I was mentally strong from the beginning because I learned that this is a normal virus, and those with a strong immune system can recover from it quickly.” (*R1*,* Student*,* Non-vaccinated)*.

Although COVID-19 was initially regarded as a lethal disease by most participants, this perception shifted in the later stages of the pandemic. Specifically, after the government launched a mass vaccination campaign and lifted strict movement restrictions, the fear of death and transmission decreased among the public. Those who were infected during this time and had been vaccinated viewed COVID-19 as a manageable disease that could be treated effectively with proper health precautions, medications, and vaccinations. Consequently, vaccinated respondents in this study reported experiencing less fear, anxiety, and stigma compared to non-vaccinated respondents. Vaccinated individuals noted that their neighbors were more supportive and empathetic because they understood that coronavirus could be managed with medication. They also observed increased support and understanding from their neighbors, relatives, and colleagues as they recognized that medication could effectively manage the coronavirus. According to one of the vaccinated respondents,

“By then, COVID-19 was seen as a treatable disease, and people understood the recovery process to be easy and quick. Severe symptoms had also reduced since 2021, so I didn’t expect to face any major challenges at that time.” *(R16*,* Student*,* Vaccinated)*.

In addition, the shift in people’s perception of COVID-19 and its impact on recovery, as well as the reduction in stigma and discrimination, became evident during our interview with a respondent who had been infected multiple times. According to him:

“The last time I got infected with COVID-19 was in September 2022. This time, I didn’t isolate myself and stayed with my whole family, including our baby. Surprisingly, no one else got infected, and my wife and family members didn’t find it scary or feel fearful. We all stayed calm and continued with our daily lives. Even though we had our baby with us, nothing harmful happened. But when I first tested positive, things were different. We took extra precautions—everyone kept their distance, and my family left my food outside my room, which I would then bring inside to avoid contact.” *(R19*,* Unemployed*,* Vaccinated)*.

#### Access to testing and mass vaccination

Participants who contracted COVID-19 during the early stages of the pandemic encountered various challenges related to coronavirus testing. These challenges included inadequate testing facilities, inaccurate test results, crowded medical environments, and non-compliance with health regulations. Their main concern was access to sufficient testing facilities. Many individuals expressed that equal access to COVID-19 testing would help normalize the situation and foster people’s trust in the healthcare system. This, in turn, would reduce the stigma associated with the disease. One of the students who lived in urban areas expressed his dissatisfaction in testing:

“The first time I went to the hospital; I wasn’t given any schedule. A staff member told me, ‘You need to make an appointment first. Come on Wednesday with a schedule.’ I went there on Sunday, but they refused to test me and told me to come back on Wednesday with an appointment. So, I left without getting the test. The following Sunday, I returned and was able to take the test.” *(R4*,* Student*,* Non-vaccinated)*.

Vaccinated individuals believed that the government’s widespread free vaccination campaigns played a crucial role in boosting public confidence regarding recovery from COVID-19. These campaigns, both nationally and globally, effectively lowered death rates, which significantly shifted public concerns and opinions about the virus. As a result, people developed a greater sense of awareness and hope, influenced by the stories of patients who had recovered. Nearly all vaccinated participants emphasized how vaccination was a key factor in changing people’s attitudes toward COVID-19, as well as in reducing stigma and discrimination. One participant shared:

“I believe the government’s vaccination program, along with other factors, played a crucial role. One thing I noticed was that, due to vaccination, the symptoms of COVID-19 were less severe. For instance, we didn’t suffer as much as people did during the early stages of the pandemic. Respiratory issues were the most painful symptom at that time, but those affected in 2021 or 2022 didn’t experience it as severely. These are the positive changes I observed, and that’s all I can speak to base on my experience.” *(R16*,* Student*,* Vaccinated)*.

#### Monitoring news media

When the pandemic first hit and there was limited information about the nature and severity of the virus, the media in Bangladesh, including both print and electronic outlets, frequently broadcasted news and conspiracy theories about the virus’s origin and its potential to cause catastrophe. This constant coverage, particularly highlighting the pandemic in leading news stories, created panic among the public in the early stages. The frequent reports on COVID-19 infections and mortality rates caused significant stress and anxiety among patients and their families. As a result, many non-vaccinated participants chose to avoid social media altogether. During an interview, one non-vaccinated frontline worker shared that, during his isolation, he chose to avoid both social media and print media in order to maintain his mental well-being. In his words:

“Every day, I was receiving news about the severity of the virus—people were dying, yet no vaccine had been invented. I realized that being on social media was gradually stressing me out, so I decided to deactivate my Facebook account to protect my mental health.**”***(R10*,* Banker*,* Non-vaccinated)*.

Throughout the pandemic, several misleading news reports and instances of mistreatment and neglect of COVID-19 patients exacerbated public fear and reinforced the stigmatization of those affected by the virus. These misrepresentations contributed to widespread anxiety, which intensified the social isolation and discrimination faced by survivors. However, as the pandemic progressed, the media shifted towards a more responsive and responsible approach, emphasizing accurate information, highlighting recovery stories, and promoting mass vaccination campaigns. This change in media strategy had a significant impact, with vaccinated patients noting that the dissemination of factual information played a vital role in alleviating stigma. One vaccinated respondent, a faculty member, emphasized:

“Media literacy and doctors’ opinions helped mitigate stigma. Additionally, the government’s successful implementation of a completely free vaccination program was a key factor in reducing the death rate and alleviating the fear surrounding COVID-19 patients” *(R20*,* faculty*,* vaccinated).*

This shift in the media’s role, as described by the respondent, reveals how the strategic use of accurate information and public health campaigns can transform the public’s perception of COVID-19 and its survivors. By focusing on recovery stories and offering clear, accessible health guidance, the media helped reduce the negative stereotypes and fear surrounding the virus.

## Discussion

This research provides a comprehensive examination of the stigma and discrimination faced by both non-vaccinated and vaccinated COVID-19 survivors in Bangladesh, analyzing data from two distinct phases of the pandemic. By examining the experiences of these two distinctive groups, the study identified three overarching themes and twelve sub-themes that reflect the evolving nature of stigma and discrimination over time. These comparative analysis reveals differences and similarities between the two groups, highlighting the broader psycho-social context of stigma and discrimination. While numerous studies have investigated the manifestations of stigma faced by COVID-19 survivors within healthcare and medical settings [15, 30, 31, 33, 34, 36, 41], this study shifts the focus to exploring the implications of stigma and discrimination in broader social contexts. The findings reveal that socio-cultural contexts and structural policies play a crucial role in shaping the differing experiences of stigma and discrimination faced by vaccinated and non-vaccinated COVID-19 survivors in Bangladesh. We argue that combating such stigma requires culturally sensitive policies, inclusive decision-making, and proactive public education. While measures like marking homes with red flags and poorly communicated lockdowns intensified fear and exclusion, initiatives such as mass vaccination and empathy-driven media campaigns proved effective in mitigating stigma when aligned with socio-cultural contexts.

The research highlights key factors of stigma and discrimination experienced by both groups, which could be addressed to reduce stigma and promote the mental well-being of survivors through policies and practices aimed at enhancing social and community support. The study identifies new sources of stigmatization that emerged during the COVID-19 pandemic, including the fear of death, infection, coronavirus testing, and public health measures such as lockdowns, public announcements, and the placement of red flags on infected individuals’ houses. Building on previous academic work, this study confirmed that the fear of death and infection were significant factors contributing to social stigma against individuals with COVID-19 [26]. However, non-vaccinated and vaccinated survivors experienced these factors differently. Due to the extensive coverage of COVID-19-related deaths on social media and television, as well as the highly contagious nature of the virus, non-vaccinated individuals experienced higher levels of fear, anxiety, avoidance, and stigma compared to vaccinated individuals. Similar research has shown that COVID-19-related deaths can intensify fear, panic, and anxiety in individuals leading to the stigmatization of COVID-19 patients in other contexts [35, 67]. However, this study challenges findings from Bor et al. [23] and Don C. Des Jarlais et al. [41], which highlight stigmatization of non-vaccinated individuals by vaccinated peers in Western countries, illustrating the context-dependent nature of stigma dynamics. Furthermore, the study reveals that both vaccinated and non-vaccinated COVID-19 survivors, especially frontline workers expressed heightened concerns about transmitting the virus to their families, resulting in self-imposed isolation and increased feelings of stigmatization [23, 41]. These findings resonate with other studies, which document the severe stigma faced by healthcare providers during the pandemic [36].

The study further highlights the unintended consequences of public health measures implemented during the COVID-19 pandemic in Bangladesh, such as lockdowns, public announcements, and the marking of survivor’s homes with red flags, which inadvertently exacerbated stigma, particularly among non-vaccinated survivors. While these measures were pivotal in controlling viral transmission, their lack of cultural sensitivity and contextual alignment often resulted in heightened social marginalization and economic precarity, especially for working-class and marginalized populations in settings like Bangladesh. In rural and urban contexts where daily labor is essential for subsistence, restrictive measures disrupted livelihoods and reinforced stigmatization tied to socioeconomic and occupational vulnerabilities. This dynamic discouraged health-seeking behaviors, including testing, as individuals feared social exclusion—paralleling findings in Malaysia, where Chew et al. reported similar stigma-driven concealment of illness [16]. Comparative evidence from a multinational study further demonstrates how stringent public health measures amplified social polarization and stigma in countries such as Mongolia, India, and the United States [5], while a systematic review by SeyedAlinahi et al. highlights the pervasive stigmatizing effects of prolonged quarantine measures globally [68]. Another systematic study by Derrer-Merk et al. [45] highlights the negative impact of protective health measures, such as confinement and self-isolation, on the emotional well-being of older adults during lockdowns, leading to increased anxiety, depression, and loneliness. These findings underscore the critical need for public health interventions that are not only epidemiologically effective but also culturally and contextually attuned, employing inclusive communication strategies and minimizing harm to vulnerable populations to ensure social equity and trust in health systems.

Gender emerged as a significant factor in the experience of stigma during the pandemic, with female participants reporting heightened stigmatization compared to their male counterparts. This gendered experience was compounded by societal norms and patriarchal expectations which heightened the challenges faced by women. Many women in the study chose to conceal their positive status from neighbors and relatives to avoid judgment, with some even opting to avoid testing altogether. In Bangladesh, women, often seen as the bearers of family honor and primary caregivers, endured intensified judgement for contracting the virus, as they were perceived to jeopardize household wellbeing. This led many to conceal their health status or avoid testing altogether, fearing gossip, ostracism or accusation of irresponsibility like this study. The lack of privacy in testing centers and economic dependence on male family members added another layer of complexity, as women risked or abandoned or reduced access to household resources if stigmatized. Moreover, unlike men, who often had greater social and economic mobility to navigate these challenges, women faced social exclusion, marital strain, and a precarious balancing between safeguarding their health and preserving their social standing. These findings align with research from Kashmir, where women faced greater stigma due to their gender identity during the pandemic and as a result, they avoid testing [69]. Furthermore, studies from the United States and other regions have also [15] documented how the fear of stigmatization drives individuals to avoid COVID-19 testing, contributing to underreporting and hindering public health efforts [7, 17]. Addressing these issues requires privacy-focused healthcare services, targeted anti-stigma campaigns, financial and social support for women, and initiatives to challenge entrenched gender norms, ensuring equitable health outcomes during future crises.

The study extensively documents the social and psychological dimensions of discrimination experienced by survivors during their quarantine periods, revealing a range of stereotype and harmful behaviors including negative comments, denial, rude interactions from neighbors, and social exclusion. These stigmatizing experiences were not only socially isolating but also contributed significantly to the deterioration of both mental and physical health among survivors. Such findings are consistent with global research on COVID-19-related stigma, which underscores the widespread nature of social marginalization and discrimination during the pandemic. In the context of Bangladesh, for example, studies have shown that individuals who contracted COVID-19 were often subject to ostracism and exclusion from their communities, perpetuating cycles of stigmatization fueled by misinformation and fear [16, 26, 40, 70]. In a similar vein, Chew et al. [16] documented how COVID-19 patients and their families were subjected to isolation, labeling, and other forms of discriminatory behavior, which exacerbated the psychological toll on affected individuals. Furthermore, broader research corroborates these findings, as seen in Gupta et al. [71] in India and Taylor et al. [72] in the United States, where COVID-19 stigma disproportionately affected marginalized groups, reinforcing pre-existing social inequities. A systematic review also emphasized that stigmatization during pandemics often manifests through social exclusion, deepening societal polarization and adversely impacting mental health [68]. These global patterns signifies the pervasive and damaging effects of COVID-19-related stigma, suggesting that addressing this issue requires not only the implementation of public health measures but also a concerted effort to challenge negative stereotypes, promote empathy, and foster supportive community networks. Moreover, this study further suggests engaging community-based organizations and grassroots networks to provide localized support to infected individuals and their families which can reduce the sense of isolation and fear of social exclusion.

The comparative analysis of stigma between vaccinated and non-vaccinated COVID-19 survivors highlights the pivotal role of vaccination in mitigating stigma, with vaccinated individuals reporting significantly lower levels of social avoidance and discrimination from families, neighbors, and colleagues. This finding aligns with SeyedAlinahi et al. [73], which documented greater stigma among non-vaccinated survivors, while vaccinated participants attributed their reduced stigma to the widespread vaccination campaign that bolstered their confidence in immunity and recovery. Similar studies by Don C. Des Jarlais [23] in the US. and Daoud LJ et al. [74] in Jordan further demonstrate how vaccination alleviated stigma, reducing social exclusion and fear. Unlike the anti-vaccination protests in some Western countries [43], Bangladesh exhibited a high public willingness to receive the vaccine, which gradually alleviated the stigma associated with COVID-19. This eagerness can be attributed to Bangladesh’s prior success in vaccination campaigns, and effective government outreach efforts that fostered public trust. Additionally, the cultural value of community solidarity in Bangladesh, combined with the government’s commitment to equitable vaccine access, helped mitigate fear and social stigmatization. However, a gap remains in the literature regarding the correlation between increasing vaccination rates and stigma reduction, suggesting an important area for future research to further understand the interplay between vaccination efforts, social acceptance, and stigma in pandemic contexts.

Our study offers key recommendations for reducing stigma and discrimination, drawing from participant feedback that can inform health policymakers and government officials in the development of effective strategies to combat stigma during pandemics. A central theme in our research is the critical role of social support and connectivity in promoting the mental well-being of COVID-19 patients. Given the imperative of maintaining physical distance during the pandemic, virtual communication emerged as a vital tool for enabling patients to stay connected with their friends and family, thus providing emotional support. Both vaccinated and non-vaccinated patients highlighted the significance of these social networks and connectivity in helping them manage stress and maintain confidence in the face of a challenging situation. Conversely, participants reported that strict isolation, particularly from family members, exacerbated their distress, underscoring the importance of family ties in emotional well-being. The study further revealed that vaccinated survivors experienced lower levels of stress, anxiety, and stigma compared to their non-vaccinated counterparts. This difference was attributed to the emotional and social support they received from family, friends, and neighbors and the vaccination campaign, which helped mitigate the negative psychological effects of quarantine and isolation. In line with these findings, our previous study on frontline workers in Bangladesh also emphasized the importance of social, organizational, and religious support as coping strategies to recover during the pandemic [37]. This finding resonates with a broader body of research [75–78], which has highlighted the significance of social support and connectivity in the recovery process from infectious diseases in various context. For instance, Pahwa et al. [79] and Mahapatra et al. [80] in India found that social networks and connectivity were crucial for coping with illness, especially due to the cultural emphasis on interdependence in the healing process. Similarly, Yang et al. [76] emphasized the vital role of social support in navigating the challenges of the lockdown in China. These findings, along with our own, underscore social support and connectivity as universal components of well-being [81, 82] during a health crisis, highlighting the need for future theoretical exploration.

Besides, our findings contrast with studies from other cultural settings, where different coping mechanisms have been identified. For example, research in Greece by Argyriadis et al. suggests that self-isolation was an important strategy for managing stigma [83], while in Malaysia, Chew et al. identified the role of religious leaders as pivotal in helping individuals cope with the psychological burden of stigma [16]. While the author’s previous research emphasized the role of spirituality as a coping mechanism for COVID-19 survivors [25], the current findings did not explore the impact of religious leaders in combating stigma. In religiously-oriented societies like Bangladesh, spiritual guidance from religious leaders often plays a crucial role in the healing process, particularly during self-isolation. Therefore, public health campaigns should incorporate imams and religious leader to deliver sermons and community talks that emphasize empathy, social solidarity, and the moral obligation to support pandemic survivors. Future research could examine how religious leaders contribute to reducing stigma and promoting recovery during pandemics, shedding light on how spiritual authority, in conjunction with community support, can help mitigate the psychological effects of such public health crises.

The availability of effective medical treatment for COVID-19, coupled with widespread vaccination campaigns, has been instrumental in alleviating the fear, anxiety, and stigma associated with infected individuals. Ensuring the accessibility and efficiency of coronavirus testing and immunization is crucial in encouraging individuals to seek medical care without the fear of social exclusion or discrimination. The government should ensure privacy in healthcare by establishing confidential testing and treatment services, including home-based care options, and implementing digital platforms for test registration and result delivery, with a particular focus on supporting women and marginalized groups to reduce stigma associated with in-person visits.

This study, alongside previous research, highlights the central role of media in shaping public perceptions during pandemics, particularly the need to regulate the dissemination of misinformation and rumors. The spread of inaccurate information through news outlets and social media can exacerbate the stigmatization of COVID-19 patients, impeding public health efforts. Studies such as those by Don C. Des Jarlais et al. [23] and Abdelhafiz et al. [84] have shown how media exposure can both heighten stigma by amplifying fears and reduce it through responsible, fact-based reporting. For instance, in the U.S., media coverage during the early phases of the pandemic contributed to heightened stigma towards COVID-19 patients [23], while in some societies, such as those observed by Abdelhafiz et al., media responsibility played a role in reducing stigma and stress [84]. Similarly, Janouskova et al. [15] demonstrated the effectiveness of mass media campaigns in mitigating stigma against healthcare professionals in the Czech Republic. In the context of Bangladesh, controlling media narratives becomes even more critical, as the country’s social fabric—marked by strong familial ties and collective identity—can exacerbate the negative impacts of misinformation. The amplification of stigma through media, especially in a society where community and religious networks play a pivotal role in social life, can lead to further marginalization of COVID-19 patients and healthcare workers, deterring individuals from seeking necessary care. Therefore, the Bangladesh government should initiate nationwide public education campaigns in partnership with media outlets to counter misinformation, promote fact-based narratives, regulate fear-driven content, and showcase positive recovery and vaccination stories, fostering a stigma-free environment.

### Strength and limitations

There were a few limitations to this comparative qualitative case study. The small number of participants restricts the generalizability of the findings, though generalizability is not the primary goal of thematic analysis or case study research, which prioritize trustworthiness. A potential bias exists in the selection process, as participants were limited to those who consented to be interviewed. In the initial stages, non-vaccinated COVID-19 survivors were more willing to share their experiences, but as the pandemic progressed recruiting an equal number of vaccinated participants for balanced comparisons became challenging. In addition, the inability to conduct face-to-face interviews due to pandemic restrictions further limited the depth of insights into participants’ experiences. Furthermore, this study did not focus on a particular social group, which may have reduced its capacity to address group-specific nuances of stigma and discrimination. Moreover, differences in policies and vaccination rates across regions likely influenced participants’ perceptions during the interviews, introducing variability in the data. Also, while the study’s findings are valuable for addressing stigma and discrimination against pandemic survivors, their applicability to other cultural or social contexts may be limited. Despite these limitations, this study makes a significant contribution to the field of public health and comparative qualitative research. Prior to this study, the authors did not come across any qualitative comparative case study that investigated the unique experiences of COVID-19 survivors who were vaccinated and those who were not, specifically focusing on their quarantine periods during the pandemic. Thus, this study could serve as a guide for future researchers to conduct more qualitative comparative case studies in other contexts, aiming to gain insights into the experiences of both vaccinated and non-vaccinated COVID-19 survivors. Furthermore, future studies can expand on this research to explore the various factors and manifestations of COVID-19 related stigma and discrimination in Bangladesh, as well as evaluate the effectiveness of any interventions aimed at reducing such stigma and discrimination.

## Conclusion

Effective public health policy should consider how and why both vaccinated and non-vaccinated COVID-19 survivors suffer various forms of stigma and discrimination in different social settings. This is particularly important to know during a pandemic because stigmatized individuals may face dire conditions while receiving medical treatment and care, which can deteriorate their mental and physical health. This comparative qualitative case study bridges this gap by exploring the underlying process of stigma and discrimination for both vaccinated and non-vaccinated COVID-19 survivors in Bangladesh. Results showed that several initiatives (e.g., lockdown, shut down, miking, strict isolation, and quarantine measures) were taken by the government during the initial phase of the pandemic to reduce the spread of the Coronavirus, which caused fear and anxiety amongst the survivors and triggered the stigmatization process. Therefore, healthcare practitioners and policymakers should consider socio-psychological circumstances and cultural contexts when implementing public health measures. Although such measures may have been successful in other contexts, in Bangladesh, they often led to negative consequences such as heightened fear and anxiety among the general population, loss of jobs, and the social avoidance of stigmatized individuals and their families ultimately affecting the well-being of COVID-19 survivors. To address these challenges, reducing fear and anxiety should prioritized as part a robust public health strategy. Additionally, adopting the WHO’s WICD decision-making tool [85] could significantly enhance patient’s recovery by promoting holistic care, reducing stigma, integrating services, and fostering health equity.

Furthermore, this research further revealed that vaccination, social support, and connectivity were very crucial aspects for both vaccinated and non-vaccinated survivors in reducing the stigmatization process. The study also revealed that because of the mass vaccination program, normalized perceptions about coronavirus, and media exposure, vaccinated individuals claimed that they experienced less stigma and discrimination than non-vaccinated individuals. Future researchers should conduct more comparative studies on COVID-19 survivors to better understand the effectiveness of mass vaccination programs in reducing stigma and discrimination in other context. This current study is significant for healthcare workers and policymakers in Bangladesh in developing effective pandemic management and control strategies. By understanding the socio-psychological and cultural intricacies of stigmatization for both vaccinated and non-vaccinated COVID-19 survivors, this study provides substantial insights into developing policies, actions, and awareness programs to minimize future pandemic-related stigma and discrimination for the vulnerable population.

## Electronic supplementary material

Below is the link to the electronic supplementary material.

Supplementary Material 1

## Data Availability

The datasets used and/or analysed during the current study are available from the corresponding author on reasonable request.
