# Supplementary material for: Comparative study of stigma and discrimination among vaccinated and non-vaccinated COVID-19 survivors in Bangladesh
Source: BMC Infect Dis. 2025 Mar 10;25:338. doi: 10.1186/s12879-025-10734-8 (PMC11895379; doi:10.1186/s12879-025-10734-8)
Supplement: Supplementary file 1 — Supplementary Material 1 [file 12879_2025_10734_MOESM1_ESM.docx]

**LIVED EXPERIENCES OF COVID-19 POSITIVE PATIENTS IN BANGLADESH**

**Probable Interview Guideline for Interviewee**

**Interviewer Introduction – READOUT**

Hello. I am …….., and Principal Investigator of this research project. I, along with our team (introduction is given in the annexure), interviewed citizens of Bangladesh who have been infected with COVID-19 but have been cured already. This study aims to explore the lived experiences of COVID-19-positive patients in Bangladesh during Quarantine. It will mainly try to explore the psycho-social experiences of COVID-19-positive patients who are now negative and spending everyday life.

**Confidentiality Clause - READOUT**

You have been chosen purposively for a particular reason. Your personal information for this study will be kept confidential so no one will know your name/address or that you participated. If you agree to participate, note that it is completely voluntary, and you can stop at any time without penalty.

If you agree to participate, please try to cooperate with us and share your valuable experiences, which may take 40-60 minutes.

| S1 | **Would you like to continue with this study?** | | |
| --- | --- | --- | --- |
|  | Yes | 1 | THANK AND CONTINUE |
|  | No | 2 | THANK AND CLOSE |

| **Section A: Demographic Information of the Respondents** | | |
| --- | --- | --- |
| Name of the Respondent |  | |
| Mobile Number |  | |
| Gender |  | |
| Age |  | |
| Religion |  | |
| Educational Qualification |  | |
| Marital Status |  | |
| Occupation |  | |
| Address | Union/Municipality |  |
|  | Upazilla/Thana |  |
|  | District |  |
| Earning Member | Key Earning Member |  |
|  | Sub-Earning Member |  |
|  | Not Employed |  |

1. **Tell me, what was your life before being positive?**

**[Information about earlier lifestyle and following health regulations]**

**Probe:**

- What was your everyday lifestyle before getting infected?
- Where did you frequently visit before sickness?
- Did you follow the health restrictions declared by the Bangladesh Government regularly?
- (Like wearing a Mask, maintaining social distance, washing hands with Soap/hand wash, hand sanitizer, etc.)

1. **How did you know that you have become positive?**

**[Information about earlier symptoms, Testing Procedure]**

**Probe:**

- What was your earlier symptom?
- Were you eager to do the test? If not, why?
- Where did you go for testing?
- Testing duration (Sample to Result)
- Testing Cost (Fee, Transport Cost, Medium/Broker)

1. **When you have been informed that you are positive, how were your initial feelings? [Information about Initial Psychological feelings after being Positive]**

**Probe:**

- How was your mental condition after hearing the news?
- Whom did you first think after hearing this?
- Did you get frightened? Why (yes/no)?
- Did you get anxious about your future?
- Did you become disappointed and hopeless?

1. **Did law enforcement agencies take any initiatives regarding your illness? What was that? Did it affect you?**

**[Information about law-enforcement agencies' initiative and social vulnerabilities]**

**Probe:**

- Lockdown
- Emergencies, etc.
- Affect my psychological state.
- Make me socially vulnerable to the community

1. **Then, what did you do for treatment and recovery?**

**[Information about treatment and isolation]**

**Probe:**

- Did you receive treatment at the hospital/ home?
- What were your experiences taking health services from Hospital/family services?
- Did you isolate yourself from everyone?

1. **Now tell me, your experiences of quarantined period?**

**[Psycho-Social Experiences during Quarantine]**

**Probe:**

- What were your experiences after being isolated from family?
- Did you affect others?
- How was your mental condition?
- What did you think during this period?
- How much supportive your family members were during this period?
- How did your relatives and neighbors support you during this period?
- Did they fear you? If yes, then why?
- Did they come to visit you?
- Did you often become frustrated by seeing their behavior?
- Did you face social harassment/stigmatization during this period?
- Does this affect you socially? How?

1. **How did you cope with this situation?**

**[Coping Mechanism]**

**Probe:**

- Need any Counseling?
- Creative work, e.g., writing, watching movies, reading books, drawing, etc.
- Praying to ALLAH/God
- Involved in other recreational activities.

1. **Now tell me, what changes come in your life after being COVID-19 positive?**

**[Psychological, Social, Economic, and other changes after Covid-19]**

**Probe:**

- Loss of jobs/ Impact on business/ Farming, etc.
- Social
- Psychological
- Religious
- Outlook

1. **Do you have any suggestions for the government and the community about this pandemic?**

**[Suggestions to government and Community Members]**

**Thank You very much for your cordial assistance.**
